# Effect of branched-chain amino acid-enriched nutritional supplementation on interferon therapy in Japanese patients with chronic hepatitis C virus infection: a retrospective study

**DOI:** 10.1186/1743-422X-9-282

**Published:** 2012-11-22

**Authors:** Yumiko Nagao, Takumi Kawaguchi, Tatsuya Ide, Michio Sata

**Affiliations:** 1 Department of Digestive Disease Information & Research, Kurume University School of Medicine, Kurume, Fukuoka, 830-0011, Japan; 2 Division of Gastroenterology, Department of Medicine, Kurume University School of Medicine, Kurume, Fukuoka, 830-0011, Japan

**Keywords:** Branched-chain amino acids (BCAA), Interferon (IFN), Hepatitis C virus (HCV), Standard IFN therapy, Low-dose long-term IFN therapy

## Abstract

**Background:**

The aims of this study were to evaluate the effects of nutritional supplementation with branched-chain amino acids (BCAA) with zinc component (Aminofeel®) on adherence to and outcome of therapy in patients treated with interferon (IFN) for chronic hepatitis C and cirrhosis and to determine whether to recommend the supplement.

**Methods:**

In this retrospective study, 51 patients who received IFN therapy were investigated among 203 consecutive patients who visited our hospital and were advised regarding the potential benefit of taking Aminofeel®. Each patient was free to choose whether to purchase and take Aminofeel®.

**Results:**

Twenty four patients (group 1-A) took Aminofeel® during standard IFN therapy and 13 (group 1-B) did not. Low-dose, long-term IFN (maintenance) therapy, mainly peglated (Peg)-IFN alpha 2a, was administered to 14 patients who were difficult to treat, because of no effect or harmful side effects with standard IFN therapy, and who had advanced liver fibrosis. Among the 14, 11 patients (group 2-A) took Aminofeel® and 3 (group 2-B) did not. The prevalence of obesity was significantly higher (P=0.04) in group 1-A than in group 1-B. The rate of adherence to IFN therapy was higher in group 1-A (83.3%) than in group 1-B (53.8%, P=0.05). There were no significant differences between the two groups in the rates of sustained virological response (SVR) to IFN therapy. According to multivariate analysis, two factors, SVR and intake of Aminofeel®, were associated with successful adherence to IFN therapy. The adjusted odds ratios for these two factors were 13.25 and 12.59, respectively, and each was statistically significant. The SVR rate of maintenance IFN therapy was in 18.2% group 2-A and 0% in group 2-B.

**Conclusion:**

Our data show that BCAA intake is useful for adherence to and effect of IFN therapy for patients with chronic hepatitis C. Nutritional supplementation with BCAA seems to be useful for HCV-infected patients receiving IFN therapy because it is impossible to introduce standard treatment for all patients among Japan's aging population.

## Background

It has been estimated that between 1 and 2 million Japanese people are chronically infected with hepatitis C virus (HCV). In Japan, elderly patients are at a higher risk for hepatocellular carcinoma (HCC) but eradication of hepatitis C virus (HCV) has a reduced effect on hepatocarcinogenesis in older patients [1,2]. Therapy using interferon (IFN), an antiviral agent, reduces the rate of occurrence of HCC and improves the long-term prognosis [3,4]. In addition, long-term pegylated (Peg) IFN therapy reduces the incidence of HCC in patients with hepatitis C and cirrhosis [5].

The current standard therapy for chronic HCV infection is a combination of Peg-IFN alpha and ribavirin (RBV). Although this therapy can achieve a sustained virological response (SVR) in approximately half of treated individuals, it is associated with significant gastrointestinal, hematological, and psychiatric side effects [6-8]. The various complications of IFN therapy are common reasons for discontinuation of treatment of HCV [9,10].

Decreases in serum levels of branched-chain amino acids (BCAA) are often seen in patients with chronic liver diseases and these decreases lead to a decline in the production of albumin and detoxification of ammonia. Therefore, BCAAs are used for the treatment of hypoalbuminemia and hepatic encephalopathy [11,12]. More recently, it was reported that BCAA could restore impaired IFN signaling and inhibit HCV replication under conditions of malnutrition [13].

BCAA are available as a pharmaceutical agent and are indicated for "improvement of hypoalbuminemia in patients with decompensated liver cirrhosis who have hypoalbuminemia (< 3.5 g/dL) despite adequate dietary intake". For that reason, in Japan, the health insurance system is not available for administration of BCAA agent to patients with hepatitis C and compensated cirrhosis who receive IFN treatment. We developed a BCAA zinc component nutritional supplement (Aminofeel®, Seikatsu Bunkasya Co. Inc. Tokyo, Japan), which is not a drug, after performing clinical testing on patients with hepatitis C. On March 1, 2007, Seikatsu Bunkasya Co released Aminofeel®. A dose of Aminofeel® contains 5.0 mg zinc and 3200.0 mg BCAA [14]. Our previous clinical trial in 2006 showed that Aminofeel® is a useful therapeutic nutritional supplement for improvement of insulin resistance, hypoalbuminemia and taste disorders and showed efficacy and safety in a post-marketing surveillance study of all HCV-infected patients [14-17].

This retrospective study was conducted to assess the value of Aminofeel® in IFN therapy for patients with chronic hepatitis C and cirrhosis. The aim of the analysis was to determine the therapeutic effects and rate of viral eradication in patients treated with IFN therapy and to determine whether to recommend supplementation with Aminofeel®.

## Results

### Effects of BCAA-enriched supplement and standard IFN therapy

We compared the characteristics of group 1-A and group 1-B (Table 1). The prevalence of obesity was significantly higher (P=0.04) in group 1-A than group 1-B.

**Table 1:** Characteristics of the study population 1 (standard IFN therapy, n=37)

		**Group 1-A with intake of Aminofeel**		**Group 1-B without intake of Aminofeel**		**P value**
No. subjects	n	24		13		
Sex	male/female	9/15		6/7		NS
Age	(mean ± SD), years	62.3 ± 5.5		57.5 ± 12.6		NS
Age range	years	50 - 74		26 - 75		
IFN therapy for the first time/retreatment	n	14/10		9/4		NS
Liver diseases	CH-C	16	66.7%	11	84.6%	NS
	CH-C & CH-B	1	4.2%	1	7.7%	NS
	CH-C & post HCV-related HCC	2	8.3%	0	0.0%	NS
	LC-C	1	4.2%	0	0.0%	NS
	LC-C & post HCV-related HCC	4	16.7%	1	7.7%	NS
Liver diseases	Only CH	17	70.8%	12	92.3%	NS
	LC or post HCC treatment	7	29.2%	1	7.7%	NS
HCV genotype	1b	19	79.2%	9	69.2%	NS
	2a	3	12.5%	4	30.8%	NS
	2b	2	8.3%	0	0.0%	NS
HCV RNA level	High	20	83.3%	11	84.6%	NS
	Low	4	16.7%	2	15.4%	NS
Genotype·HCV RNA level	1b·High	17	70.8%	9	69.2%	NS
	others	7	29.2%	4	30.8%	NS
Extrahepatic manifestations	Diabetes millitus (positive %)	8	33.3%	4	30.8%	NS
	Hypertensiton (positive %)	6	25.0%	2	15.4%	NS
	Hyperlipidemia (positive %)	2	8.3%	0	0.0%	NS
	Oral lichen planus (positive %)	0	0.0%	1	7.7%	NS
	Hyperthyroidism (positive %)	0	0.0%	1	7.7%	NS
	Hypothyroidism (positive %)	3	12.5%	1	7.7%	NS
Total of Aminofeel intake (g)	mean ± SD	1000.0 ± 673.0		0		<0.0001
BMI	mean ± SD	23.3 ± 3.8		22.0 ± 1.7		NS
Obesity (BMI ≥25 kg/m^2^)	n (%)	7	29.2%	0	0.0%	0.04
RBC (×10^4^/μL)	mean ± SD	438.8 ± 45.9		450.2 ± 41.7		NS
Hb (g/dL)	mean ± SD	13.7 ± 1.2		14.1 ± 1.5		NS
PLT (×10^4^/μL)	mean ± SD	13.3 ± 4.1		15.5 ± 7.0		NS
WBC (μL)	mean ± SD	4412.5 ± 1348.9		4853.8 ± 1066.6		NS
PT (%)	mean ± SD	86.5 ± 17.9		92.8 ± 6.4		NS
AST (U/I)	mean ± SD	59.6 ± 25.9		57.6 ± 36.5		NS
ALT (U/I)	mean ± SD	64.7 ± 46.9		73.2 ± 59.2		NS
LDH (U/I)	mean ± SD	208.9 ± 42.0		199.5 ± 54.1		NS
gamma GTP (U/I)	mean ± SD	38.4 ± 19.5		41.8 ± 33.1		NS
ChE (U/I)	mean ± SD	206.4 ± 87.1		262.8 ± 71.1		NS
TP (g/dL)	mean ± SD	7.7 ± 0.5		7.6 ± 0.4		NS
Alb (g/dL)	mean ± SD	4.00 ± 0.5		4.17 ± 0.2		NS
T.Bil (mg/dL)	mean ± SD	1.0 ± 0.3		0.8 ± 0.2		NS
FBS (mg/dL)	mean ± SD	117.5 ± 43.7		99.7 ± 14.9		NS
HbA1c (%)	mean ± SD	5.4 ± 0.7		5.7 ± 1.0		NS
TC (mg/dL)	mean ± SD	167.9 ± 26.6		170.4 ± 13.4		NS
AFP (ng/dL)	mean ± SD	8.1 ± 11.5		5.5 ± 3.0		NS
IRI (μU/mL)	mean ± SD	31.3 ± 35.6		13.2 ± 7.1		NS
HOMA-beta		216.2 ± 11.9		203.8 ± 258.8		NS
HOMA-IR		11.9 ± 21.4		3.3 ± 2.0		NS
Zn (μg/dL)	mean ± SD	68.1 ± 11.5		73.9 ± 11.4		NS
Course of IFN therapy	Peg-IFN alpha 2b/RBV	21	87.5%	8	61.5%	
	Peg-IFN alpha 2a/RBV	0	0.0%	1	7.7%	
	Peg-IFN alpha 2a monotherapy	0	0.0%	3	23.1%	
	Peg-IFN alpha 2a monotherapy - (change) - Peg-IFN alpha 2a/RBV	0	0.0%	1	7.7%	
	Peg-IFN alpha 2a monotherapy - (change) - IFN beta	1	4.2%	0	0.0%	
	Peg-IFN alpha 2b/RBV - (change) - Peg-IFN alpha 2a monotherapy - (change) - Peg-IFN alpha 2b/RBV	1	4.2%	0	0.0%	
	IFN alpha	1	4.2%	0	0.0%	
Continuation of IFN therapy	Successful continuation	20	83.3%	7	53.8%	0.05
	Discontinuation	4	16.7%	6	46.2%	0.05
Effect of IFN therapy	SVR	12	50.0%	9	69.2%	NS
	Non-SVR	12	50.0%	4	30.8%	NS

HCV, hepatitis C virus; CH-C, chronic hepatitis C; CH-B, chronic hepatitis B; LC-C, liver cirrhosis type C; HCC, hepatocellular carcinoma; RBC, red blood cell; Hb, hemoglobin; PLT, platelets; WBC, white blood cell; PT, prothrombin time; AST, aspartate aminotransferase; ALT, alanine aminotransferase; LDH, lactate dehydrogenase; gamma GTP, gamma-glutamyltransferase; ChE, cholinesterase; TP, total protein; Alb, albumin; T.Bil, total bilirubin; FBS, fasting blood glucose; TC, total; cholesterol; IRI, immunoreactive insulin; Zn, zinc; SVR, sustained virological response; NS, not significant; IFN, interferon; RBV, ribavirin.

The rate of adherence to IFN therapy was 83.3% (20/24) in group 1-A patients, and 53.8% (7/13) in group 1-B and was significantly higher (P=0.05) in group 1-A. The average total intake Aminofeel® in group 1-A was 1,000.0 ± 673.0 g. The most effective of the treatments in group 1-A and B was combination therapy with Peg-IFN alpha 2b and RBV. There were no significant differences in the SVR rates of IFN therapy between the two groups.

Table 2 documents the reasons for discontinuation of therapy. Adverse events and abnormal laboratory tests were documented in 6 and 3 patients, respectively. One patient was lost because of relocation. As adverse events, encephalopathy, exacerbation of hypothyroidism, fundal hemorrhage, pneumonia, anorexia, arthralgia, and sleeplessness were reported. The abnormal laboratory tests were elevated transaminases and HCV RNA levels. Seven of the 10 patients (70%) who discontinued IFN therapy did not achieve SVR. The HCV genotype of two patients was 2a, among three patients who achieved SVR in spite of discontinuing therapy.

**Table 2:** Reasons for discontinuation of standard IFN therapy (n=10)

	**Age**	**Sex**	**Liver disases**	**Extrahepatic manifestations**	**HCV genotype**	**HCV RNA level**	**Course of IFN therapy**	**Reasons for discontinuation of IFN therapy**	**Effect of IFN therapy**
Group 1-A with intake of Aminofeel (n=4)	70	F	LC-C & post HCV-related HCC	None	1b	High	Peg-IFN alpha 2b/RBV	Encephalopathy	Non-SVR
	67	F	CH-C	Hypertenstion, hyperlipidemia, and hypothyroidism	1b	High	Peg-IFN alpha 2b/RBV	Exacerbation of hypothyroidism	Non-SVR
	58	F	CH-C	Diabetes mellitus and hypertenstion	1b	High	Peg-IFN alpha 2b/RBV	Fundal hemorrhage	Non-SVR
	66	M	CH-C	Diabetes mellitus	1b	High	Peg-IFN alpha 2b/RBV	Increased transaminase	Non-SVR
Group 1-B without intake of Aminofeel (n=6)	65	F	CH-C	None	1b	High	Peg-IFN alpha 2b/RBV	Increased HCV RNA levels	Non-SVR
	26	F	CH-C	None	2a	High	Peg-IFN alpha 2b/RBV	Relocation	SVR
	65	F	CH-C	Hypertenstion	1b	High	Peg-IFN alpha 2b/RBV	Pneumonia	Non-SVR
	59	F	CH-C	Hypertenstion	1b	High	Peg IFN alpha 2a monotherapy - (change) - Peg-IFN alpha 2a/RBV	Increased HCV RNA levels	Non-SVR
	54	M	CH-C	Diabetes mellitus	2a	Low	Peg-IFN alpha 2a monotherapy	Anorexia	SVR
	64	M	CH-C	None	1b	High	Peg-IFN alpha 2b/RBV	Arthralgia and sleeplessness	SVR

CH-C, chronic hepatitis C; LC-C, liver cirrhosis type C; HCC, hepatocellular carcinoma; SVR, sustained virological response; IFN, interferon; RBV, ribavirin.

### Multivariate analysis

All variables in the univariable analyses were included in multivariable analysis. According to multivariate analysis, two factors, SVR and intake of Aminofeel®, were associated with successful completion of IFN therapy. The adjusted odds ratios for these two factors were 13.25 and 12.59, respectively, and each was statistically significant.

### Effects of BCAA-enriched supplement and maintenance IFN therapy

We compared the characteristics of group 2-A and group 2-B (Table 3). The SVR rate of long-term IFN therapy was in 18.2% (2/11) group 2-A patients and 0% (0/3) in group 1-B. The most useful treatment in group 2-A and B was Peg-IFN alpha 2a monotherapy.

**Table 3:** Characteristics of the study population 2 (maintenance IFN therapy, n=14)

		**Group 2-A with intake of Aminofeel**		**Group 2-B without intake of Aminofeel**		**P value**
No. subjects	n	11		3		
Sex	male/female	3/8		3/0		0.02
Age	(mean ± SD), years	65.2 ± 5.9		64.3 ± 6.0		NS
Age range	years	56 - 73		58 - 70		
IFN therapy for the first time/retreatment	n	5/6		0/3		NS
Liver diseases	CH-C	1	9.1%	0	0.0%	
	CH-C & AIH	1	9.1%	0	0.0%	
	CH-C & post HCV-related HCC	1	9.1%	1	33.3%	
	LC-C	5	45.5%	0	0.0%	
	LC-C & LC-B	1	9.1%	0	0.0%	
	LC-C & post HCV-related HCC	2	18.2%	2	66.7%	
Liver diseases	Only CH	2	18.2%	0	0.0%	NS
	LC or post HCC treatmnet	9	81.8%	3	100.0%	NS
HCV genotype	1b	8	72.7%	2	66.7%	NS
	2a	2	18.2%	0	0.0%	NS
	unknown	1	9.1%	1	33.3%	NS
HCV RNA level	High	11	100.0%	3	100.0%	NS
	Low	0	0.0%	0	0.0%	NS
Extrahepatic manifestations	Diabetes millitus (positive %)	5	45.5%	1	33.3%	NS
	Hypertensiton (positive %)	6	54.5%	1	33.3%	NS
	Hyperlipidemia (positive %)	0	0.0%	0	0.0%	NS
	Oral lichen planus (positive %)	1	9.1%	0	0.0%	NS
	Hyperthyroidism (positive %)	2	18.2%	0	0.0%	NS
	Hypothyroidism (positive %)	1	9.1%	0	0.0%	NS
Total of Aminofeel intake (g)	mean ± SD	2443.6 ± 3209.3		0		<0.01
BMI	mean ± SD	24.3 ± 2.0		24.5 ± 3.1		NS
Obesity (BMI ≥ 25 kg/m^2^)	n (%)	4	36.4%	1	33.3%	NS
RBC (×10^4^/μL)	mean ± SD	402.3 ± 62.3		417.3 ± 29.5		NS
Hb (g/dL)	mean ± SD	13.0 ± 1.5		13.1 ± 1.2		NS
PLT (×10^4^/μL)	mean ± SD	9.4 ± 4.8		10.2 ± 4.9		NS
WBC (μL)	mean ± SD	3809.1 ± 1045.4		3500.0 ± 1558.8		NS
PT (%)	mean ± SD	80.7 ± 22.6		85.3 ± 10.5		NS
AST (U/I)	mean ± SD	83.9 ± 44.4		73.7 ± 8.4		NS
ALT (U/I)	mean ± SD	77.9 ± 34.9		68.3 ± 6.5		NS
LDH (U/I)	mean ± SD	238.7 ± 79.1		211.0 ± 31.2		NS
gamma GTP (U/I)	mean ± SD	57.7 ± 35.0		63.0 ± 23.4		NS
ChE (U/I)	mean ± SD	166.3 ± 98.4		188.0 ± 42.3		NS
TP (g/dL)	mean ± SD	7.4 ± 0.6		7.7 ± 0.6		NS
Alb (g/dL)	mean ± SD	3.59 ± 0.5		3.67 ± 0.1		NS
T.Bil (mg/dL)	mean ± SD	1.0 ± 0.3		1.1 ± 0.4		NS
FBS (mg/dL)	mean ± SD	112.7 ± 18.4		93.3 ± 24.0		NS
HbA1c (%)	mean ± SD	5.6 ± 0.8		5.4 ± 0.9		NS
TC (mg/dL)	mean ± SD	166.7 ± 20.8		134.5 ± 9.2		NS
AFP (ng/dL)	mean ± SD	33.7 ± 70.2		11.7 ± 3.8		NS
IRI (μU/mL)	mean ± SD	20.8 ± 15.1		15.6 ± 8.8		NS
HOMA-beta		185.7 ± 184.0		208.6 ± 69.7		NS
HOMA-IR		6.0 ± 5.3		3.9 ± 3.2		NS
Zn (μg/dL)	mean ± SD	62.8 ±10.7		67.0 ± 2.8		NS
Course of IFN therapy	Peg-IFN alpha 2a monotherapy	4	36.4%	2	66.7%	
	Peg-IFN alpha 2a/RBV - (change) - Peg-IFN alpha 2a monotherapy	4	36.4%	1	33.3%	
	IFN beta - (change) - Peg-IFN alpha 2b/RBV - (change) - Peg-IFN alpha 2a monotherapy	1	9.1%	0	0.0%	
	Peg-IFN alpha 2a monotherapy - (change) - Peg-IFN alpha 2a/RBV - (change) - Peg-IFN alpha 2a monotherapy	1	9.1%	0	0.0%	
	Peg-IFN alpha 2a monotherapy - (change) - Peg-IFN alpha 2b/RBV	1	9.1%	0	0.0%	
Effect of IFN therapy	SVR	2	18.2%	0	0.0%	NS
	Non-SVR	9	81.8%	3	100.0%	NS

HCV, hepatitis C virus; CH-C, chronic hepatitis C; LC-C, liver cirrhosis type C; LC-B, chronic hepatitis type B; HCC, hepatocellular carcinoma; AIH, autoimmune hepatitis; IFN, interferon; RBC, red blood cell; Hb, hemoglobin; PLT, platelets; WBC, white blood cell; PT, prothrombin time; AST, aspartate aminotransferase; ALT, alanine aminotransferase; LDH, lactate dehydrogenase; gamma GTP, gamma-glutamyltransferase; ChE, cholinesterase; TP, total protein; Alb, albumin; T.Bil, total bilirubin; FBS, fasting blood glucose; TC, total; cholesterol; IRI, immunoreactive insulin; Zn, zinc; SVR, sustained virological response; NS, not significant; IFN, interferon; RBV, ribavirin.

One of two subjects in whom SVR was obtained was a 58-year-old woman who suffered from HCV & hepatitis B virus (HBV)-related liver cirrhosis. The number of PLT before IFN therapy was 4.2 × 10^4^/μL. We administered IFN beta (3 million units/day for 2 week and thereafter 3 million units/week) from September 5, 2006, and changed IFN beta to Peg-IFN alpha 2b and RBV from February 8, 2007 and to Peg-IFN alpha 2a (45 μg with 2-week intervals) from July 25, 2007 to February 17, 2009.

The other was a 68-year-old woman who suffered from HCV-related liver cirrhosis and hyperthyroidism. The number of PLT before IFN therapy was 5.1×10^4^/μL. We administered her Peg-IFN alpha 2a (90 μg/week) from July 28, 2004 to July 24, 2009.

### Prevalence of extrahepatic manifestations

The prevalence extrahepatic diseases was higher in group 2 than group 1. The complications in group 1 included: diabetes mellitus (DM) (n=12, 32.4%), hypertension (n=8, 21.6%), hyperlipidemia (n=2, 5.4%), oral lichen planus (n=1, 2.7%), hyperthyroidism (n=1, 2.7%), and hypothyroidism (n=4, 10.8%). The complications in group 2 included: DM (n=6, 42.9%), hypertension (n=7, 50.0%), oral lichen planus (n=1, 7.1%), hyperthyroidism (n=2, 14.3%), and hypothyroidism (n=1, 7.1%).

## Discussion

HCC is one of the most common malignancies, especially in Japan. The most important risk factors of HCC are chronic hepatitis C and B and cirrhosis. IFN therapy has therapeutic benefits but management of therapy is required because the treatment leads to a number of side effects [6-10]. The major side effects include fatigue, influenza-like syndrome, gastrointestinal disturbances and abnormalities of laboratory tests. The rate of discontinuation of combination therapy with Peg-IFN alfa and RBV was reported to be 10-26% [6-10]. Most dose discontinuations resulted from adverse events, whereas only a minority required discontinuation due to abnormalities of laboratory tests [8]. Fried *et al.* demonstrated that a full dosing scheduling of both Peg-IFN and RBV permits 75% of early virological responders to achieve SVR, that dose reduction resulted in a fall to 67% and that discontinuation resulted in a loss of SVR, at 12% [7].

In this study, we showed that intake of a BCAA-enriched supplement is significantly associated with the ability to adhere to standard IFN therapy. Our previous clinical evaluation of the BCAA-enriched supplement, Aminofeel®, in chronic hepatitis C patients who were not receiving IFN therapy, showed improvement of fatigue, loss of appetite, jitteriness, and leg cramps [14]. Evidence produced elsewhere shows improvement of insulin resistance, hypoalbuminemia, taste disorders, ChE value, and prothrombin value [14-17]. Kawaguchi *et al.* suggested the following three reasons for improvement of subjective symptoms with BCAA intake: amelioration of hepatic encephalopathy, improvement of malnourishment by elevated tryptophan levels and increase of impaired cerebral blood flow [18-21]. Our previous study using single photon emission computed tomography (SPECT) showed decreased regional cerebral blood flow during IFN administration [22]. This phenomenon induces psychotic symptoms such as a depressive state.

We reported previously a strong association between hypoalbuminemia and mortality in a hyperendemic area (X town) of HCV infection in Japan [23]. Residents with hypoalbuminemia had a mortality of 68.0%; dramatically higher than the rate of 12.1% among residents who had normal albumin levels. Improvement of hypoalbuminaemia should be considered for improvement of prognosis.

Combination therapy with Peg-IFN and RBV is a useful strategy for patients with hepatitis C but it is impossible to introduce standard treatment for all patients because it is not adapted for abnormal biochemical findings or elderly patients. Liver-protective therapy, such as oral administration of ursodeoxycholic acid or intravenous injection of Stronger Neo-minophagen C (SNMC®), is commonly given to nonresponders to IFN therapy in Japan [24,25]. SNMC® is a glycyrrhizin preparation that has potent anti-inflammatory activity and has been used in Japan for centuries to treat allergic diseases and hepatitis. However, this agent is not considered to have any antiviral or anticancer effect [26]. We have reported the effect of IFN on alpha-fetoprotein (AFP) changes in 40 patients with chronic hepatitis C [27]. AFP was reduced significantly more in the IFN group than the SNMC group (P = 0*.*0034). Therapeutic strategies for hepatitis C, e.g., low-dose long-term IFN treatment, reduced the incidence not only of hepatocarcinogenesis but also esophageal varices during the 17-year observation period [28,29].

There are very few reports that SVR was obtained by long-term, low dose therapy for chronic hepatitis C [30]. However, in this study, we succeeded in eradicating HCV from two patients with cirrhosis using maintenance IFN treatment. HCV is frequently reported to complicate extrahepatic manifestations such as oral lichen planus, thyroid disease and DM [31]. Most of the 10 patients who discontinued standard IFN therapy had extrahepatic manifestations, and most of the 14 who received low-dose long-term IFN therapy also had such manifestations. These manifestations are reported to be exacerbated by IFN therapy and lead to discontinuation of therapy. These findings indicate the value of maintenance IFN therapy for patients with extrahepatic manifestations.

Amongst Japan's aging population, combined therapy of low-dose long-term IFN and administration of BCAA is thought to be a treatment to adopt enthusiastically. BCAA intake seems to be useful for HCV-infected patients.

## Conclusion

In conclusion, our data show that BCAA intake is useful for continuation of IFN therapy for patients with HCV chronic liver diseases. There is great variability among subjects with liver disease and, because this is not a randomized study, further investigations are needed.

## Materials and methods

A total of 203 patients with chronic liver diseases who had a checkup in Kurume University Hospital from March 1, 2007 to July 31, 2008 received advice about the therapeutic effects of, and methods of purchasing, Aminofeel®. Each patient was free to decide whether to purchase and take Aminofeel®. We excluded 15 patients whose uptake rates were below 70%.

Among the remaining 188 patients, there were 51 who received IFN therapy. Thirty five patients took Aminofeel® and 16 did not. Thirty seven patients received IFN treatment with the aim of eradicating HCV and 14 received low-dose, long-term (maintenance) IFN treatment aimed at suppression of hepatocarcinogenesis. Natural IFN alpha, IFN beta or Peg-IFN alpha 2a was selected for IFN monotherapy. Combined standard therapy of Peg-IFN alpha 2b or 2a and RBV was given for 24 to 72 weeks.

### Group 1 (standard IFN therapy)

Among the 37 patients who received IFN therapy to eradicate HCV, 24 (group 1-A) took Aminofeel® during therapy and 13 (group 1-B) did not (Table 1). Group 1-A ranged in age from 50 to 74 years, with an average of 62.3 ± 5.5 years. Group 1-B ranged in age from 26 to 75 years, with an average of 57.5 ± 12.6 years.

The diagnosis of liver disease in group 1-A included: chronic hepatitis C (n=16), chronic hepatitis C and hepatitis B (n=1), chronic hepatitis C with post-treatment of HCC (n=2), HCV-related liver cirrhosis (n=1), and HCV-related liver cirrhosis with post-treatment of HCC (n=4). Those of group 1-B included: chronic hepatitis C (n=11), chronic hepatitis C and hepatitis B (n=1), and HCV-related liver cirrhosis with post-treatment of HCC (n = 1).

The end-point was the achievement of the adherence to IFN and/or RBV therapy, defined as the receipt of ≥ 80% of scheduled IFN and/or RBV doses for ≥ 80% of the scheduled treatment period.

### Group 2 (low-dose, long-term IFN therapy)

Low-dose long-term (maintenance) IFN, usually Peg-IFN alpha 2a, was administered to 14 patients who were difficult to treat, because of no effect or harmful side effects with standard IFN therapy, and who had advanced liver fibrosis (Table 3). Among the 14, 11 patients (group 2-A) took Aminofeel® during therapy and 3 (group 2-B) did not. Group 2-A ranged in age from 56 to 73 years, with an average of 65.2 ± 5.9 years. Group 2-B ranged in age from 58 to 70 years, with an average of 64.3 ± 6.0 years.

The diagnosis of liver disease in group 2-A included: chronic hepatitis C (n=1), chronic hepatitis C and autoimmune hepatitis (n=1), chronic hepatitis C with post-treatment of HCC (n=1), HCV-related liver cirrhosis (n=5), HCV and HBV-related liver cirrhosis (n=1), and HCV-related liver cirrhosis with post-treatment of HCC (n=2). Those of group 2-B included: chronic hepatitis C with post-treatment of HCC (n=1), and HCV-related liver cirrhosis with post-treatment of HCC (n=2).

### Serological assays

All patients were tested for red blood cell (RBC), white blood cell (WBC), platelets (PLT), hemoglobin (Hb), and insulin resistance index (IRI), zinc (Zn), fasting blood glucose (FBS) and HbA1c, and for the following liver function tests: serum alanine aminotransferase (ALT), aspartate aminotransferase (AST), lactate dehydrogenase (LDH), gamma-glutamyltransferase (gamma GTP), cholinesterase (ChE), prothrombin time (PT), total bilirubin (T. Bil), total cholesterol (TC), total protein (TP), and albumin (Alb). The formula for the HOMA-IR is: HOMA-IR = FBS × fasting insulin/405. The formula for the HOMA-beta is: HOMA-beta = 360 × fasting insulin/FBS-63.

### Evaluation of liver diseases

Anti-HCV was measured using a chemiluminescent enzyme immunoassay kit (Lumipulse II HCV, Fujirebio, Tokyo, Japan). HCV RNA in serum was analyzed by the Amplicor HCV test (Roche, Tokyo, Japan) up to December 6, 2007 and by quantitative PCR assay (COBAS AMPLICOR HCV MONITOR v 2.0 Test, COBAS AmpliPrep/COBAS Taq-Man HCV Test, Roche Molecular Systems, New Jersey, US) from December 7, 2007 [32,33]. HCV genotype was determined by polymerase chain reaction assay, using a mixture of primers for the subtype, as reported previously [34]. HBsAg was assayed using a chemiluminescent immunoassay kit (Architect, HBsAg QT, Dainabot, Tokyo, Japan). All patients were examined using hepatic ultrasonography, computed tomography, or magnetic resonance imaging in order to investigate the shape of the liver and lesions occupying the liver. Liver biopsy was performed on some patients.

### Evaluation of extrahepatic diseases

IFN therapy may cause or worsen such immunological diseases or extrahepatic diseases. We examined theses diseases before IFN therapy. A diagnosis of oral lichen planus was made on the basis of clinical and/or histopathological features. Obesity was defined as a body mass index (BMI) ≥ 25 kg/m^2^. Diagnosis of type 2 DM was based on the American Diabetic Association (ADA) criteria of 1997 [35]. Hypertension was defined as a systolic blood pressure (SBP) of 140 mmHg or higher or a diastolic blood pressure (DBP) of 90 mmHg or higher, according to the criteria of JNC-VI of the International Hypertension Society [36]. Thyroid hormones such as FT3, FT4 and thyroid stimulating hormone were measured for all patients and thyroid echography examination was performed for some patients. Examination of the upper or lower gastrointestinal tract was performed on patients for whom it was deemed clinically necessary.

### Ethical considerations

The retrospective study was approved by the Ethics Committee of Kurume University on August 4, 2008 (reference number: 06046) in accordance with the Declaration of Helsinki.

### Statistical analysis

All data are expressed as mean ± standard error. Differences between the two groups were analyzed using the Mann–Whitney *U* test, Wilcoxon’s test, and Fisher’s exact test. Differences were judged significant for p ≤ 0.05 (two-tailed). P value was rounded off to two decimal places. Adjusted odds ratios were calculated using logistic regression analysis. All statistical analyses were conducted using JMP Version 6 (SAS Institute, Cary, NC, USA). The level of statistical significance was defined as 0.05.

## Abbreviations

HCV: Hepatitis C virus; CH-C: Chronic hepatitis C; CH-B: Chronic hepatitis B; LC-C: Liver cirrhosis type C; LC-B: Liver cirrhosis type B; HCC: Hepatocellular carcinoma; PBC: Primary biliary cirrhosis; IFN: Interferon; RBV: Ribavirin; SVR: Sustained virological response; PLT: Platelets; Hb: Hemoglobin; ALT: Alanine aminotransferase; AST: Aspartate aminotransferase; LDH: Lactate dehydrogenase; ALP: Alkaline phosphatase; T.Bil: Total bilirubin; TC: Total cholesterol; TP: Total protein; Alb: Albumin.

## Competing interests

The authors declare that they have no competing interests. None of author in this study holds and is applying for any patents relating to Aminofeel®. None of author has received fees, funding, or salary from Seikatsu Bunkasya Co. Seikatsu Bunkasya Co is applying for a patent for Aminofeel®.

## Authors’ contributions

YN carried out most of the data collection, designed the study, performed the statistical analysis, and drafted the manuscript. TK provided comments during manuscript preparation. TI contributed to data collections and clinical diagnosis. MS contributed to data analysis. All authors read and approved the final manuscript.
